# *bk-5* ^*214S2L*^ *,* an allelic variant of *bk-5,* as high-quality silage maize genetic resource

**DOI:** 10.3389/fgene.2025.1483839

**Published:** 2025-02-28

**Authors:** Gang Li, Jiyuan Du, Xiaohu Li, Shilin Zhuge, Shuolin Ren, Min Wu, Haoran Ma, Xinrui Guo, Ziqiang Chen, Haiping Ding

**Affiliations:** ^1^ Institute of Biotechnology, Fujian Academy of Agricultural Sciences, Fuzhou, Fujian, China; ^2^ National Key Laboratory of Wheat Breeding, College of Life Sciences, Shandong Agricultural University, Taian, China

**Keywords:** maize, brittle stalk, silage, detergent fiber, mutant

## Abstract

**Introduction:**

Stem brittleness significantly affects both yield and quality of maize.

**Methods:**

Using phenotypic identification and sequence analysis, we identified a new brittle stalk maize mutant. Furthermore, we assessed its feeding value by content determination of cellulose, hemicellulose, lignin crude fiber, starch, and protein contents.

**Results:**

Here, we identified a brittle stalk maize mutant, *bk-5* ^*214S2L*^, an allelic variant of *bk-5*. The stem brittleness of *bk-5* ^*214S2L*^ was similar to that of *bk-5*, but not identical. Unlike *bk-5*, *bk-5* ^*214S2L*^ leaves did not fall off completely and its stems did not break in windy conditions. We identified a missense mutation (C>T) in the fifth exon of the candidate gene *Zm00001d043477*, resulting in an amino acid change from serine to leucine at position 214. A significant reduction in cell wall thickness in the leaf veins and stems of *bk-5* ^*214S2L*^ compared with the inbred line RP125. Among the major cell wall components in stems and leaves, total cellulose, hemicellulose, and lignin were lower in *bk-5* ^*214S2L*^ than in RP125. We also evaluated the application value of *bk-5* ^*214S2L*^ silage and found that the detergent fiber contents of *bk-5* ^*214S2L*^ stems were significantly reduced compared with RP125, while the crude fiber, starch, and protein contents remained unchanged. The reduced tannin content improved the palatability of the silage for livestock.

**Conclusion:**

Overall, *bk-5* ^*214S2L*^, an allelic variant of *bk-5*, is a high-quality genetic resource for breeding forage and grain-feed maize.

## 1 Introduction

Maize (*Zea mays* L.) silage, an important livestock feed, is a major source of energy and rich in water-soluble carbohydrates, and can be used as a major silage ingredient (Garon et al., 2006; Kaplan et al., 2016; Uher et al., 2019; Serva, 2024). It is heavily used in silage production and is one of the main sources of silage because of its low cost, high yield, nutrient richness, palatability, and digestibility (Sheaffer et al., 2006; Guyader et al., 2018; Salama, 2019; Maize silage | Feedipedia, n.d.). The yield and quality of silage maize are key indicators of its merit as forage (Zhao et al., 2022; Caratti et al., 2024). The global area under silage maize cultivation is about 16.8 million ha (http://www.agpm.com). The area under silage maize cultivation in Europe and United States has increased to 6.13 million ha and 2.66 million ha, accounting for 42% and 7% of total maize production, respectively (Mirzaei et al., 2023) (https://www.statista.com/statisticians/190890; https://www.feedipedia.org). Silage maize production is expected to increase in more countries. For example, China’s silage maize cultivation area is only 1.6 million ha, less than one-tenth of the *per capita* cultivation area of Europe and United States, while the production area is expected to reach 6.7 million ha by 2030 (Li et al., 2021). Although silage maize production and industrial processing and applications have developed relatively fast in China, silage maize germplasm resources of good quality are still relatively scarce (Liu et al., 2024).

From the perspectives of food efficiency and healthy consumption, the feeding capacity of ruminant livestock and the demand for high-quality roughage will increase (Mj et al., 2021). Feed yield determines the amount of dry matter available to livestock, and feed quality affects animal growth (and livestock products) by influencing their digestibility and energy intake (Khan et al., 2012; Richman et al., 2015). The quality of maize silage is determined by energy content, intake potential, and protein and mineral contents. Cellulose and hemicellulose in silage corn are important energy sources. However, the high content of lignin will reduce the digestibility of animals and affect the absorption of energy (Guo et al., 2022; Kumar Gupta and Govintharaj, 2023). Lignin, a complex aromatic polymer, forms a tightly - bound matrix with cellulose and hemicellulose within the plant cell wall structure. This intricate binding restricts the access of digestive enzymes secreted by the animal and the activity of microorganisms in the rumen or other parts of the digestive system, thereby reducing the extent to which cellulose and hemicellulose can be broken down into simpler, absorbable components (Nie et al., 2020; Silva et al., 2021). Intake potential is negatively correlated with the crude fiber (CF), acid detergent fiber (ADF), and neutral detergent fiber (NDF) contents of maize silage (Zhao et al., 2008; Zhang et al., 2018). A set of grades has been formulated by the state for the ADF and NDF contents of maize feeds: NDF content ≤45% and ADF content ≤23% is classified as grade 1 (best); NDF content ≤50% and ADF content ≤26% is classified as grade 2; and NDF content ≤55% and ADF content ≤29% is classified as grade 3 (GB/T 25882-2010).

In this study, a maize brittle stalk mutant with a variant in the candidate gene *Zm00001d043477* (B73: version 4) was identified by map-based cloning. Sequencing revealed a single-base mutation from C to T in the coding region, resulting in a change of amino acid 214 from serine (S) to leucine (L), which was named *bk-5* ^*214S2L*^. This mutant was a weak allelic variant of the brittle stalk mutant *bk-5* (Li et al., 2022). Although *bk*-*5* ^*214S2L*^ plants still showed brittleness compared with *bk-5*, they did not experience leaf breakage under extreme weather conditions. Mutations at different loci of this gene resulted in differences in plant brittleness and changes in the levels of cellulose, hemicellulose, and lignin. CF, ADF, and NDF, indicators related to silage digestion, were highly significantly reduced in *bk-5* ^*214S2L*^ compared with the maize inbred line RP125, whereas no significant differences were found in plants for starch and crude protein contents. In addition, a reduction in tannin content increased the palatability of the feed. Thus, *bk-5* ^*214S2L*^ is a high-quality genetic resource for breeding silage maize.

## 2 Materials and methods

### 2.1 Plant materials and investigation of agronomic traits

The *brittle stalk-5* ^*214S2L*^ (*bk-5* ^*214S2L*^) mutant, characterized by its fragile stems and overall plant frailty, is a weak allelic variant of the *bk-5* maize mutant (Li et al., 2022). All mutant materials were homozygous variants in the ethyl methanesulfonate (EMS)-induced mutant library of the inbred line RP125 (considered the wild type in this study). RP125 pollen grains were treated with a 0.1% EMS solution (Sigma-Aldrich, M0880) dissolved in mineral oil (Sigma-Aldrich, M8410) for 30–40 min. Subsequently, 60–70 ears were subjected to pollination to induce the mutation. Using this method, our group established an EMS mutant library in the RP125 background (Nie et al., 2021). Within this library, systematic screening for mutants exhibiting brittle culm traits led to the discovery of a stably inherited brittle culm mutant, named *bk-5* ^*214S2L*^, by successive selfing. Throughout the growth phases—from germination and seedling to nodulation, silking, and maturity—*bk-5* ^*214S2L*^ plants underwent field observations for phenotypic evaluation. At the nodulation stage, a stem-breaking experiment was performed. Upon reaching maturity, various agronomic traits, including compressive strength, plant height, spike height, spike length, spike thickness, grain dimensions, spike weight, and 100-grain weight, were measured in both the mutant and RP125. Furthermore, *bk-5* ^*214S2L*^ was crossed with Mo17 to generate F1 and F2 populations for subsequent genetic analyses and gene mapping.

### 2.2 Determination of compressive strength

Rind Penetrometer Resistance: Ten plants from each of RP125 and *bk-5* ^*214S2L*^ exhibiting comparable growth potential were selected at the nodulation stage. Using a penetrometer (YYD-1, ToPu YunNong) with a pressure plate, the rind penetrometer resistance was assessed in the middle of the third internode of the stem. The measurements were performed until the stems ruptured, capturing the peaks of resistance of plants.

Stem Bending Strength (Three-Point Bending Test, TPBT): Likewise, ten plants from each of RP125 and *bk-5* ^*214S2L*^ were selected at the nodulation stage. All stems from node 1 to node 6 of the stalk were excised with a knife. Subsequently, the internodal length of the third internode of the stem was measured, along with its diameter. The stem peak strength was measured using a YYD-1 stem strength tester. The stem bending strength was calculated as follows: M_*max*_ = PL/2, where M_*max*_ represents the bending strength, P denotes the stem peak strength, and L signifies the internodal length (Hou et al., 2022). During the measurement, we set up six biological replicates.

### 2.3 Histochemical staining and determination of cell wall contents

To visualize cellulose and hemicellulose within the cell wall, leaf veins from the third internode of the aboveground stem of RP125 and *bk-5* ^*214S2L*^ plants were selected at the jointing (elongation) stage. In addition, leaf blades at the spike were included in the study. Using an ultrathin tissue slicer, internodes and leaf veins were precisely sectioned into 60-μm transverse sections. Subsequently, staining was conducted using cellulose staining solution (zinc chloride iodide; Leagene, DP0406) for 2 min. The sections were then observed under a light microscope (Leica, Wetzlar, Germany). For lignin observation, the third internode was similarly sectioned and stained using resorcinol (Ookawa et al., 2014). The sections were fixed in a 1% resorcinol/alcohol solution (v/v) for 2 min (Sigma, 79330), followed by color development by the addition of 18% HCl solution (Hossain et al., 2012). The stained sections were covered with a coverslip and observed under a light microscope (Leica) (Li et al., 2022).

Stems, along with leaves, from RP125 and *bk-5* ^*214S2L*^ plants at the jointing stage were used for cellulose content determination. A cellulose measurement kit (Solarbio, BC4280) was used, and the steps included cell wall material (CWM) extraction, CWM drying, and cellulose extraction. Absorbance analysis, coupled with a chromogenic reaction of concentrated sulfuric acid with anthrone, facilitated the quantification. For lignin measurement, a lignin measurement kit (Solarbio, BC4200) was used. The internodes were dried and ground, followed by extraction and determination of lignin content using perchloric acid and glacial acetic acid. Air-dried or oven-dried samples were ground into powder and sieved for hemicellulose content determination. Hemicellulose hydrolysis included detecting reducing sugars by a characteristic absorption peak at 540 nm, which enabled the quantitative assessment using the hemicellulose content determination kit (Solarbio, BC4440). During the measurement, we set up three biological replicates and six experimental replicates.

### 2.4 Paraffin sectioning and scanning electron microscope observations

At the jointing stage, stems (from the third internode above ground) of RP125 and *bk-5* ^*214S2L*^ plants were collected on ice.

#### 2.4.1 Microscopic analysis of stem structure

The stems were fixed with a 70% FAA (formaldehyde, alcohol, acetic acid) fixative at 4°C for 24 h. The subsequent procedures involved dehydration, clarification, wax embedding, sectioning, deparaffinization, and staining. Finally, the prepared tissue sections were examined under a light microscope (Nie et al., 2021).

#### 2.4.2 SEM analysis

The stems were fixed in a 2.5% glutaraldehyde solution (w/v, Solarbio, P1126). Sample pretreatment was conducted using the experimental platform of the State Key Laboratory of Shandong Agricultural University. Ethanol concentration gradients (5%, 10%, 20%, 30%, 50%, 70%, 80%, 90%, 100%) were sequentially applied for 30 min each to eliminate the water content. Subsequently, isoamyl acetate concentration gradients (25% isoamyl acetate and 75% anhydrous ethanol, 50% isoamyl acetate and 50% anhydrous ethanol, 75% isoamyl acetate and 25% anhydrous ethanol, 100% isoamyl acetate) were used to displace ethanol, with overnight incubation in 100% isoamyl acetate. The prepared intersegmental sheets were subjected to critical point drying, sputter plating, and observation under SEM (Sigma 500, Zeiss) at various magnifications (×30, ×150, ×300). To determine cell wall thickness, 10 values were measured at ×300 magnification from 20 neighboring cells around the lumen of the vascular bundle closest to the thick-walled layer in both RP125 and *bk-5* ^*214S2L*^. Each value was halved to obtain the wall thickness of each cell. Likewise, three fields of view were counted to derive 3 × 10 values, and the means ± standard deviation (SD) values were calculated. Data was analyzed using ImageJ software (rsb.info.nih.gov/ij) (Li et al., 2023). During the measurement, we set up six biological replication.

### 2.5 RNA extraction and quantitative real-time PCR analysis

Using the RNA extraction kit (TIANGEN, DP419), total RNA was carefully extracted from distinct tissues of both RP125 and *bk-5* ^*214S2L*^ at the nodulation stage. Subsequently, cDNA was synthesized by reverse transcription with the Evo M-MLV Reverse Transcription Kit II (AG, AG11711) and Evo M-MLV Plus cDNA Synthesis Kit (AG, AG11615). For gene expression analysis, qRT-PCR was conducted in an Applied Biosystems thermal cycler. The SYBR Premix Ex TaqII (Tli RNase H Plus) Kit (TaKaRa, RR820A) was used for qRT-PCR assays, ensuring accurate and reliable quantification of gene expression levels. RNA Denaturation is 65°C for 5 min. Reverse Transcription is 45°C for 30–60 min. Enzyme Inactivation is 70°C for 10 min. Pre-denaturation is 95°C for 3 min. Denaturation: 95°C for 10–30 s. Annealing: 55°C for 30 s. Extension is 72°C for 30 s. Cycles number are 40 (Nie et al., 2021). Primer sequences are provided in Supplementary Table S3. During the measurement, we set up six experimental replicates.

### 2.6 Evaluation of silage maize indicators

#### 2.6.1 Determination of detergent fiber contents

Mature dry maize plants were pulverized using a pulverizer and passed through a 40-mesh sieve. The processed samples were stored in sealed bags for future use. Approximately 1.0 g (m) of the samples were weighed, placed into polyester filter bags with known mass (m_1_), and hermetically sealed using a thermoplastic machine. Each sample was subjected to three replicates. In a 3L beaker, 2 L of neutral detergent was introduced and brought to a boil. Subsequently, 20 filter bags were immersed in the boiling solution for 60 min. Deionized water was replenished during boiling to maintain a consistent solution concentration. After boiling, the filter bags were rinsed, dried at 105°C to a constant weight in an aluminum box, and immediately covered to prevent moisture. After cooling, the bags were weighed (m_2_). In a subsequent step, 2 L of acid detergent was added to the same beaker and brought to a boil. The 20 filter bags previously used were placed in the boiling solution for 60 min, with periodic replenishment of deionized water. After boiling, the filter bags were rinsed, dried at 105°C to a constant weight in an aluminum box, and immediately covered to prevent moisture. After cooling, the bags were weighed (m_3_) (Nazli et al., 2019). The fiber content was calculated as follows:

$$\text{NDF content }\left(\%\right)=\left(m_{2}-m_{1}\right)\;/\;m\times 100\%$$

$$\text{ADF content }\left(\%\right)=\left(m_{3}-m_{1}\right)\;/\;m\times 100\%$$

#### 2.6.2 Determination of crude protein content

Total nitrogen content was quantified with the A33 flow analyzer. The samples were dried at 85°C over approximately 1 week. Subsequently, 0.5 g of the dried samples was precisely weighed and added to 12 mL of concentrated sulfuric acid along with 5 g of copper flakes as a catalyst. Digestion was performed using an automated high-temperature digestion system (420°C for 90 min), and the mixture was allowed to cool naturally. The digested solution was then carefully transferred to 50-mL volumetric flasks. To ensure optimal conditions for analysis, the concentration of concentrated sulfuric acid in the digested solution was carefully adjusted to 4%. The total nitrogen content was determined using the A33 flow analyzer (Liu et al., 2024).

#### 2.6.3 Determination of starch content

The starch content of plants was assessed with a starch content assay kit (Solarbio, BC0700). Approximately 0.03 g of the sample was accurately weighed and crushed in a mortar and pestle. Soluble sugars and starch were effectively isolated from the sample using 80% ethanol. The starch thus obtained underwent further decomposition into glucose by acid hydrolysis. The glucose content was determined by the anthrone colorimetric method. Subsequently, the starch content was calculated based on the measured glucose content (Khan et al., 2023).

#### 2.6.4 Tannin content analysis

To determine tannin content, a tannin content assay kit (Solarbio, BC1390) was used. The samples were dried to a constant weight, finely crushed, and sieved through a 30–50 mesh. Approximately 0.1 g of the sieved sample was weighed and mixed with 2 mL of the extract solution. The mixture was centrifuged for 10 min at 70°C in a water bath at 12,000 rpm. Subsequently, the supernatant was carefully extracted, and the sample was adjusted to 2 mL with the extract solution. The extract solution was adjusted to zero, and standards were prepared by diluting to concentrations of 25, 12.5, 6.25, 3.125, 1.5625, and 0.78125 nmol/mL with the extract solution. After thorough mixing and shaking for 5 min, centrifugation was performed at 13,000 g for 20 min at room temperature. The supernatant was then used to measure the absorbance at 275 nm in a UV spectrophotometer (preheated for 30 min). A standard curve was established based on the concentration and absorbance values of the standards. The tannin content of the sample was calculated using the standard curve.

#### 2.6.5 Measurement of soluble sugar content

Following the method of Ibrahim et al. (2012), 50 mg of the crushed sample was carefully placed in a 15-mL centrifuge tube. Subsequently, 10 mL of 80% ethanol was added to the tube. The samples were cooled in a water bath at 80°C for 30 min. After centrifugation at 4,000 r/s for 3 min, the supernatant was extracted. To this supernatant, 10 mg of activated charcoal was introduced, and the mixture was once again cooled in a water bath at 80°C for 30 min to eliminate any stray colors. The solution was then subjected to filtration. The soluble sugar content was determined using the anthrone-sulfate method, with measurements taken at an absorbance of 620 nm.

## 3 Results

### 3.1 Phenotypic characterization of *bk-5* ^*214S2L*^

**FIGURE 1:**
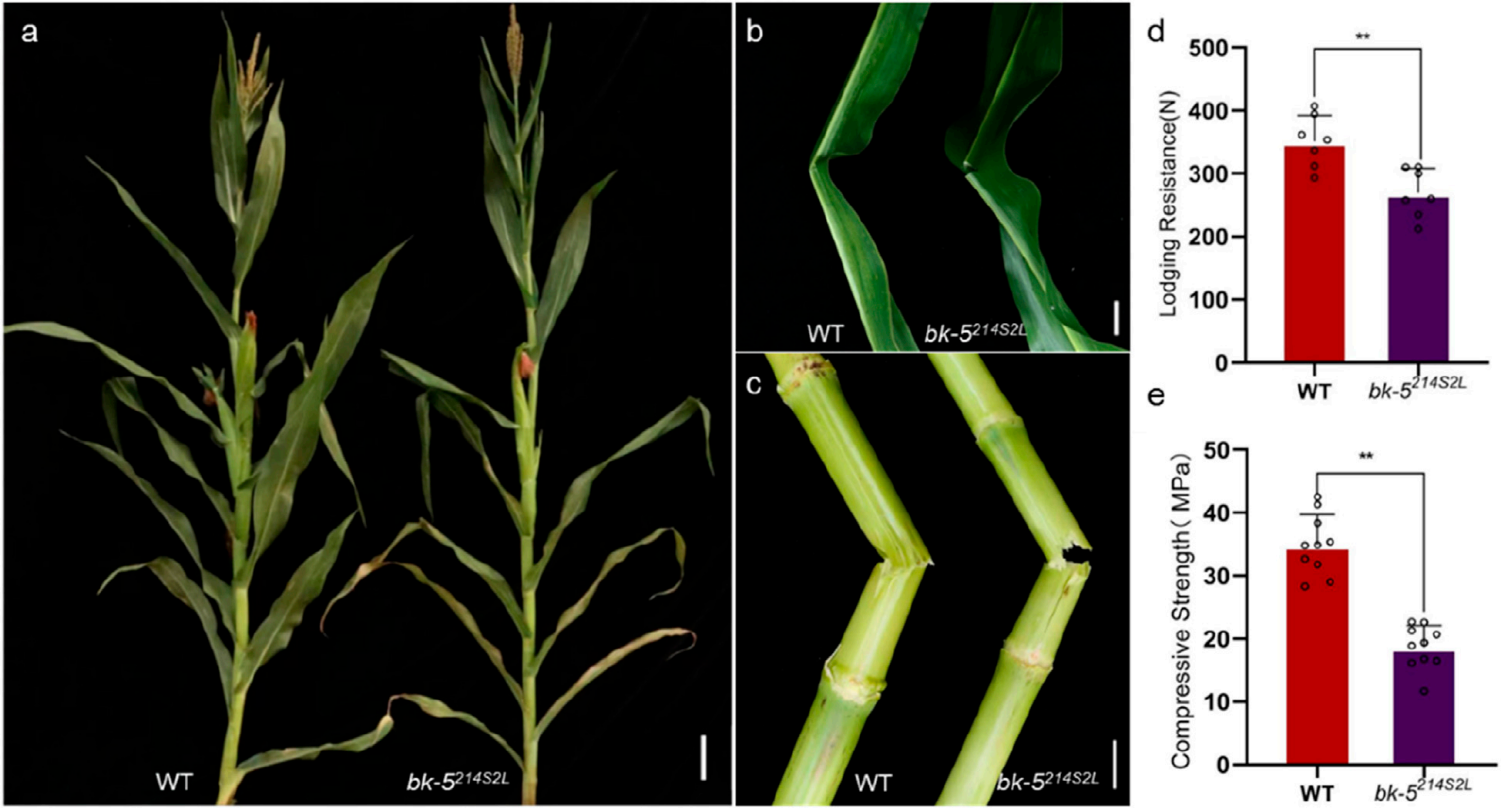
Characterization of *bk-5* ^*214S2L*^. **(A)** Phenotypic comparison between RP125 (WT, left) and *bk-5* ^*214S2L*^ (right) at maturity. Scale bar: 10 cm. **(B)** Comparison of leaf breakage between RP125 (left) and *bk-5* ^*214S2L*^ (right) at the jointing stage. Scale bar: 1 cm. **(C)** Comparison of stem breakage between RP125 (left) and *bk-5* ^*214S2L*^ (right) at the jointing stage. An easily broken stem of *bk-5* ^*214S2L*^ was observed, and both stems broke with the same force. Scale bar: 5 cm. **(D)** Determination of stem lodging resistance of RP125 (left) and *bk-5* ^*214S2L*^ (right) at maturity. **(E)** Determination of stem compressive strength of RP125 (left) and *bk-5* ^*214S2L*^ (right) at maturity (n = ten plants). Values are presented as means ± standard deviation (SD) (Student’s t-test; **p < 0.01 indicates a significant difference between RP125 and *bk-5* ^*214S2L*^).

A genetically stable maize brittle stalk mutant was identified from the EMS mutant library (Nie et al., 2021). This mutant was named *bk-5* ^*214S2L*^ (*brittle stalk-5* ^*214S2L*^) based on its “brittle and breakable” phenotype. Phenotypic characterization of *bk-5* ^*214S2L*^ showed that mutant plants were brittle from the three-leaf stage, with brittle stems till even the nodulation stage and maturity. Field phenotyping of the mutant showed no change in plant shape compared with RP125 at either seedling or nodulation stage (Figure 1A). In stem-breaking experiments in which the same portion of the stem at the jointing stage was bent with the same force, *bk-5* ^*214S2L*^ stems broke completely, with neat and smooth breakage, whereas RP125 stems remained attached and did not undergo complete breakage (Figures 1B, C). Therefore, the stem lodging resistance and stem compressive strength were measured (Figures 1D, E), and a significant decrease was observed in both, with a 39.2% decrease in stem lodging resistance and a 23.3% decrease in stem compressive strength. To determine whether the mutation of the candidate gene affected the yield traits of plants, the spike length, spike weight, 100-grain weight, grain length, grain width, and grain thickness were measured at maturity. All yield traits were significantly lower in mutant plants than in RP125 plants (Supplementary Figure 1). Thus, the mutation of the candidate gene affected not only the plant’s stem bending resistance but also its yield.

### 3.2 Histochemical staining observations

**FIGURE 2:**
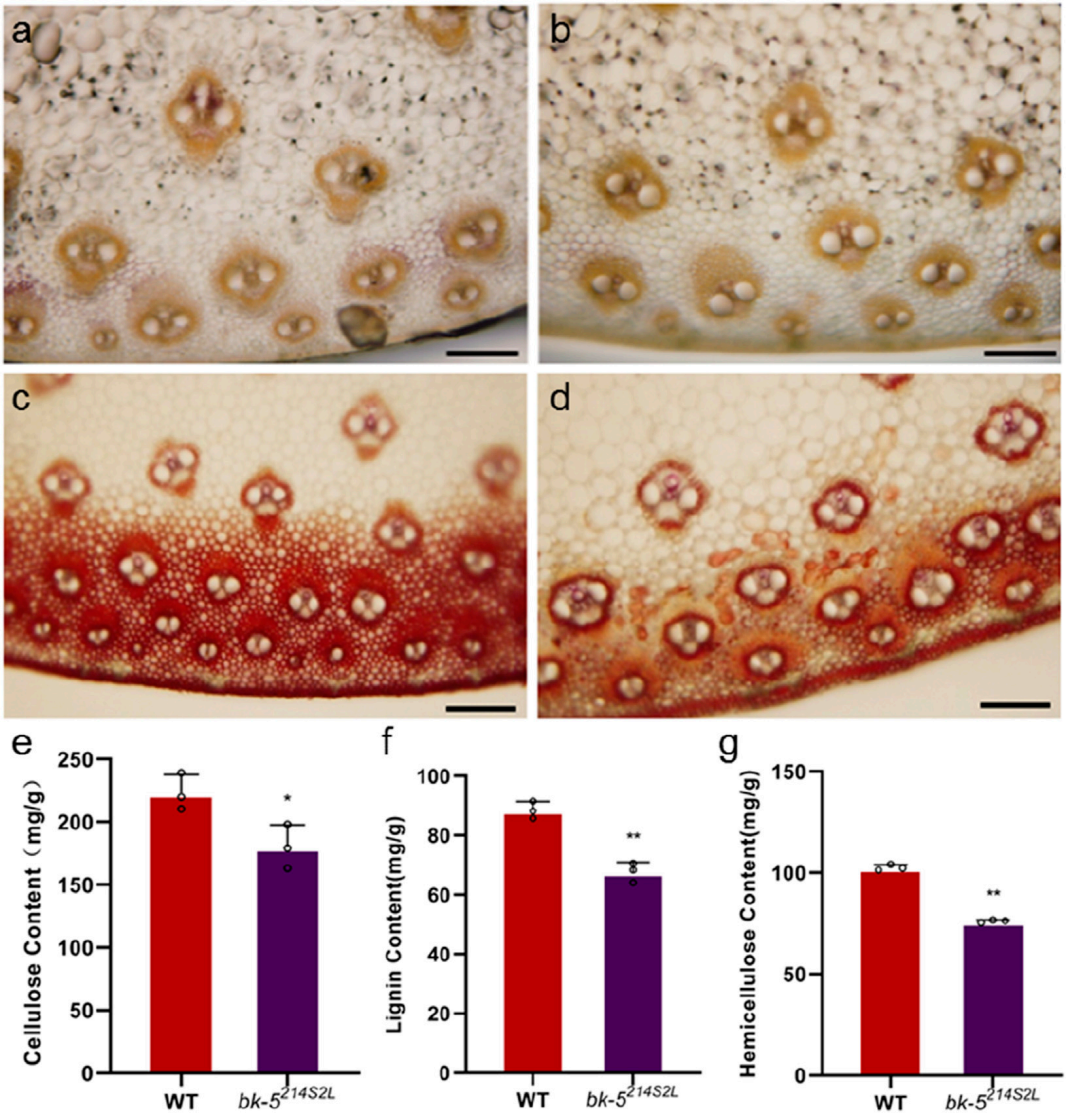
Histochemical staining and cell wall contents of stems. **(A, B)** Staining of cellulose and hemicellulose of RP125 (wild type, WT) stem **(A)** and *bk-5* ^*214S2L*^ stem **(B)** by zinc chloride iodide solution. The darker the color means the higher the cellulose and hemicellulose contents. **(C, D)** Staining of lignin of RP125 stem **(C)** and *bk-5* ^*214S2L*^ stem **(D)** by phloroglucinol solution. Pink represents lignin deposition, and the darker the pink, the higher the lignin content. **(E)** Cell wall cellulose content of *bk-5* ^*214S2L*^ and RP125 stems. **(F)** Cell wall lignin content of *bk-5* ^*214S2L*^ and RP125 stems. **(G)** Cell wall hemicellulose content of *bk-5* ^*214S2L*^ and RP125 stems. All samples were from plants at the jointing stage, and the third internode above ground was sectioned. Values are presented as means ± SD (Student’s t-test; *p < 0.05 indicates a significant difference between RP125 and *bk-5* ^*214S2L*^). Each group consisted of three biological replicates.

The reduced stem mechanical strength suggests that the cell wall composition of mutant plants was altered. Therefore, we performed histochemical staining and determined the contents of cellulose, hemicellulose, and lignin in the stems and leaf veins (aboveground third node) and leaf blades at the spike of *bk-5* ^*214S2L*^ and RP125 plants at the nodulation stage (Figure 2; Supplementary Figure 2). In the staining results, the color change of cellulose and hemicellulose staining was lesser in *bk-5* ^*214S2L*^ stem sections than in RP125 stem sections, indicating that the difference in cellulose and lignin content of the stem was small, whereas the staining colors of cellulose and hemicellulose in leaf vein sections were significantly lighter, indicating that the cellulose and hemicellulose contents of the leaf veins were significantly reduced (Figures 2A-D). The results of the cellulose content assay showed that the stem cellulose content was 223.2 mg/g in RP125 and 180.3 mg/g in *bk-5* ^*214S2L*^, which was significantly reduced by 19.22% compared with RP125 (Figure 2E). The leaf cellulose content was 357.3 mg/g in RP125 and 251.3 mg/g in *bk-5* ^*214S2L*^, which was highly significantly reduced by 29.67% compared with RP125 (Supplementary Figure 2E). Lignin staining of stem sections of *bk-5* ^*214S2L*^ was significantly lighter, indicating that the lignin content was significantly reduced in the mutant, whereas lignin staining of leaf vein sections was less variable, indicating no significant difference in the lignin content of leaf veins. The lignin content assay showed no significant change in the leaf lignin content of *bk-5* ^*214S2L*^ compared with RP125, but the stem lignin content was highly significantly reduced by 23.56% (Supplementary Figure 2F; Figure 2F). The results of the hemicellulose content assay showed that the stem hemicellulose content was 102.7 mg/g in RP125 and 76.2 mg/g in *bk-5* ^*214S2L*^, which was highly significantly reduced by 25.8% compared with RP125 (Figure 2G). The leaf hemicellulose content was 192.6 mg/g in RP125 and 174 mg/g in *bk-5* ^*214S2L*^, which was significantly reduced by 9.66% compared with RP125 (Supplementary Figure 2G). These results suggest that the mutant gene plays an important role in the biosynthesis of cell wall components. The results also indicate that the main reason for the low compressive strength of *bk-5* ^*214S2L*^ was the reduced contents of cell wall components.

### 3.3 Changes in thick-walled tissues and cell wall structure of *bk-5* ^*214S2L*^ plants

**FIGURE 3:**
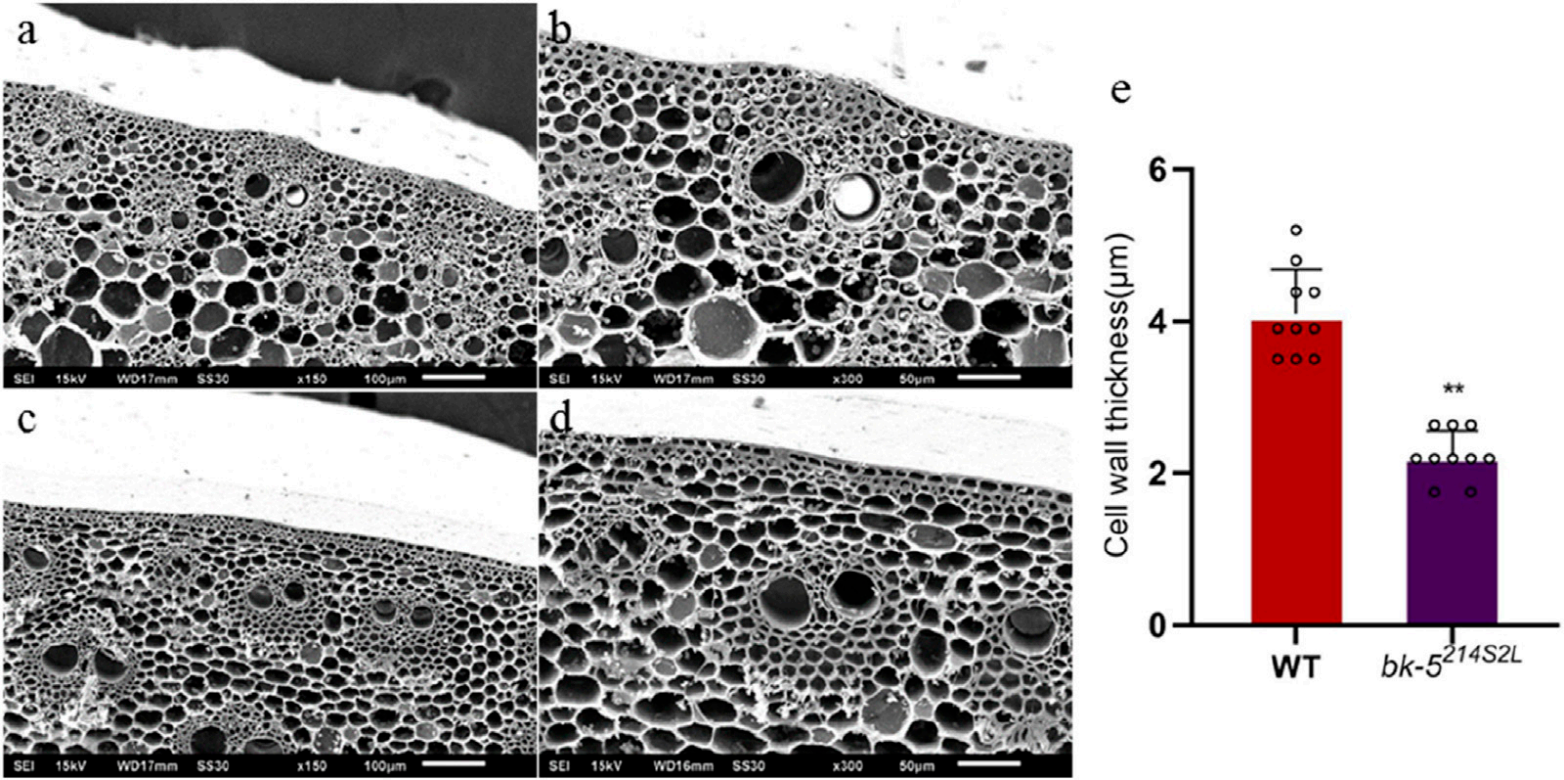
Stem cross-section under scanning electron microscopy. **(A, B)** Cross-section of RP125 stem. **(C, D)** Cross-section of *bk-5* ^*214S2L*^ stem. Different magnifications (×150, ×300) are shown. **(E)** Cell wall thickness of RP125 and *bk-5* ^*214S2L*^ vascular bundle cells near the sclerenchyma. Ten values of RP125 and *bk-5* ^*214S2L*^ were determined, and data was analyzed using ImageJ software. Values are presented as means ± SD (Student’s t-test; **p < 0.01 indicates a significant difference between RP125 and *bk-5* ^*214S2L*^).

Cellulose is an important cell wall component, and changes in its content affect the formation and structure of the cell wall. Therefore, we investigated the anatomical characteristics of thick-walled cells in the leaf veins and stems of RP125 and *bk-5* ^*214S2L*^ plants. Changes in the cell wall structure may be an important factor contributing to the reduced compressive strength of *bk-5* ^*214S2L*^ plants. Transverse sections of leaf veins showed that the thickness of thick-walled tissues (number of cell layers) in the leaf veins of *bk-5* ^*214S2L*^ was significantly reduced, becoming fragile and irregular (Supplementary Figure 3). SEM of thick-walled tissues showed a significant reduction in wall thickness of cells around the vascular bundles in the leaf veins of *bk-5* ^*214S2L*^ compared with RP125, and a significant reduction in cell wall thickness of thick-walled tissues was observed in *bk-5* ^*214S2L*^ stems (Figures 3A–D). The wall thickness of cells around the vascular bundles was quantified using ImageJ software, and *bk-5* ^*214S2L*^ cell wall thickness was highly significantly reduced by 47.5% compared with RP125 (Figure 3E). Longitudinal sections of leaf veins showed that the thickness of thick-walled tissues (number of cell layers) in the leaf veins of *bk-5* ^*214S2L*^ was significantly reduced and irregular (Supplementary Figure 4). Therefore, reduced cell wall thickness and the thinning of thick-walled tissues (reduction in the number of cell layers) may be responsible for the reduced mechanical strength.

### 3.4 Genetic analysis and map-based cloning of *bk-5* ^*214S2L*^

**FIGURE 4:**
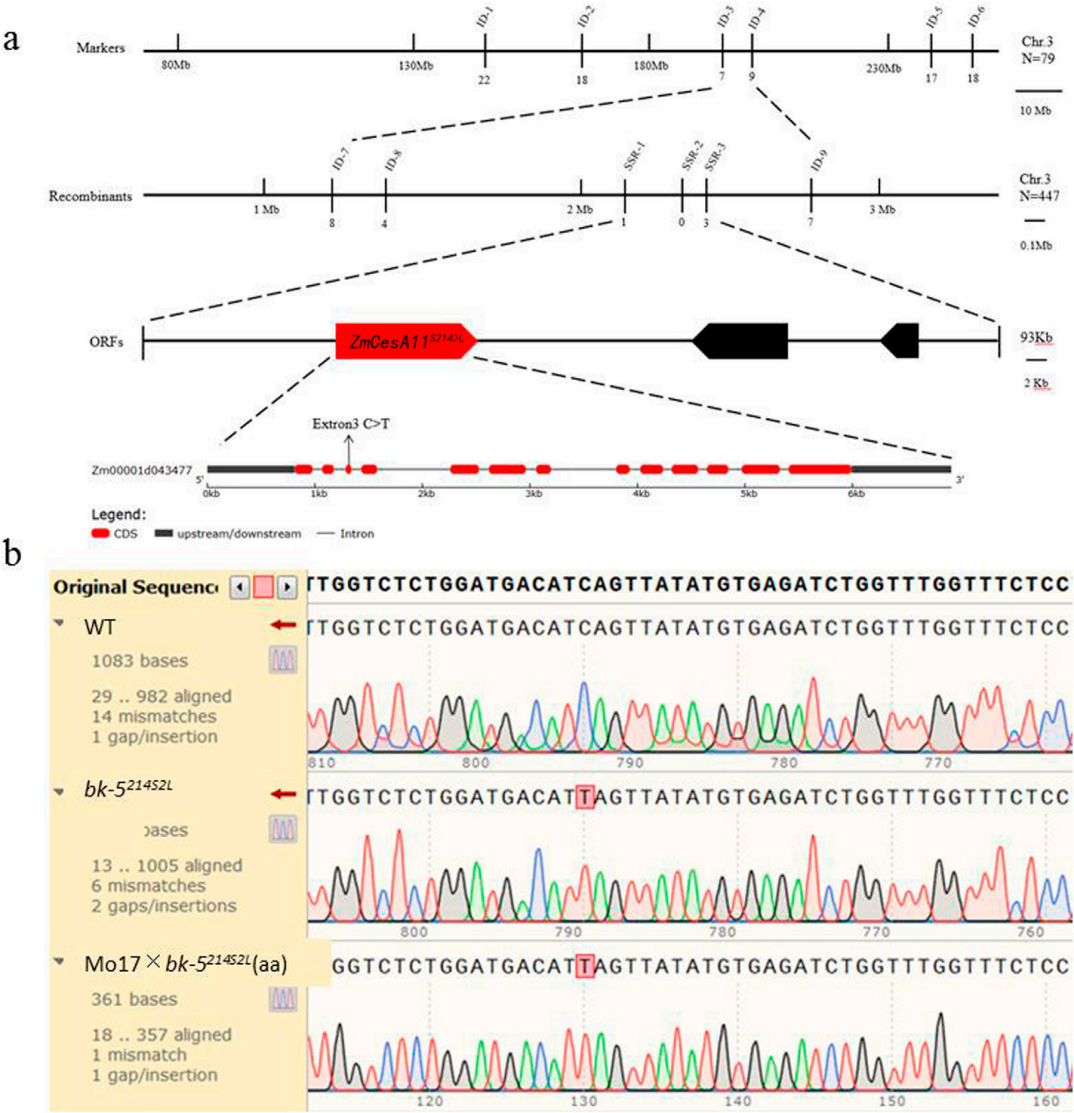
Map-based cloning and identification of the mutated gene of *bk-5* ^*214S2L*^ using Mo17 × *bk-5* ^*214S2L*^ F2 segregation populations. **(A)** Map-based cloning of *bk-5* ^*214S2L*^. *bk-5* ^*214S2L*^ was preliminarily mapped between the flanking markers ID3 and ID4 on chromosome 3 using Mo17 × *bk-5* ^*214S2L*^ F2 segregation populations, with an estimated length of 16.3 Mb (n = 79 individuals with zebra leaf phenotype). *bk-5* ^*214S2L*^ was finely mapped between molecular markers SSR1 and SSR3, with an estimated length of 93 kb (n = 447 individuals with the brittle stalk phenotype). The target region contained three ORFs based on the genome sequence data. A mutation from C to T on the last exon of gene *Zm00001d043477* (*T001*) led to a mutation of the 214th amino acid serine (S) to leucine (L). ORFs, open reading frames; N, number of F2 plants. **(B)** Sequence alignment of RP125 and *bk-5* ^*214S2L*^ candidate genes.

The F1 generation was obtained by crossing *bk-5* ^*214S2L*^ (male parent) with Mo17 (female parent), and the F2 segregation population was obtained by self-crossing. The phenotypes of the F2 generation were counted and were normal:mutant = 3:1, in accordance with Mendel’s law of inheritance, which suggested that the phenotypes were controlled by a single recessive mutation (Supplementary Table 1). Thereafter, 15–20 leaves of normal and brittle stalk plants from the F2 generation were taken, mixed in equal quantities, and DNA was extracted to make the dominant and recessive pools for bulk segregation analysis. More than 200 pairs of SSR markers covering 10 chromosomes of maize were used to screen RP125, Mo17, F1, and the dominant and recessive pools of RP125. SSR markers were used to screen RP125, Mo17, F1, and the dominant and recessive pools for polymorphism. As a result of the screening, markers ID-1 and ID-3 located on chromosome 3 were strongly associated with the target genes (Supplementary Figure 5). Based on the two SSR markers obtained, 160 F2 brittle culm monocotyledons were amplified, and the monocotyledon exchange rate was calculated, and it was initially determined that the localization interval was located upstream of marker ID-4 (Supplementary Figure 5). To determine the location of the *bk-5* ^*214S2L*^ mutant gene, new InDel markers were developed and screened upstream of marker ID-4 using the maize Mo17 reference genome information as well as RP125 sequence data (Supplementary Table 2). All brittle culm monocotyledons in the F2 generation population were screened with these markers, the bands were counted, and the exchange rate was calculated. The target region was narrowed down step by step to 93 kb between SSR1 and SSR3, and the locus was mapped (Figure 4A).

According to the fine-mapping results, combined with the reference genome gene annotation information, three ORFs were found in the 93-kb region between SSR1 and SSR3 (Figure 4A). Thus, the corresponding specific primers were designed according to the three ORFs, and the three genes were amplified and sequenced using DNA of RP125 and *bk-5* ^*214S2L*^ as templates to detect the mutation sites. The sequencing results showed that only *Zm00001d043477* had a single-base mutation from C to T (Figure 4A), and the sequencing peak map showed that the SNP site was a single peak, indicating that the sequencing results were credible. For verification, 20 leaves of brittle culms were selected from the F2 generation plants of Mo17 × *bk-5* ^*214S2L*^, and DNA was extracted and mixed in equal amounts to make a cryptic mixed pool. The obtained SNP locus was amplified and sequenced using the mixed pool DNA as a template. The sequencing results showed that this locus in the recessive pool was also a pure mutation, and the sequencing peaks were single peaks (Figure 4B), which indicated that *Zm00001d043477* was the mutant gene of *bk-5* ^*214S2L*^. Analysis of the Gramene database revealed that the *Zm00001d043477* gene was 4,464 bp in length, with the longest transcript of 3,248 bp encoding 535 amino acids, which was functionally annotated as encoding the 11 subunits of cellulose synthase. A single-base mutation changed its 214th amino acid from serine (S) to leucine (L); thus, it was named *ZmCesA11* ^*S214>L*^.

### 3.5 Differential expression analyses of *candidate gene and related* CesA *genes*

**FIGURE 5:**
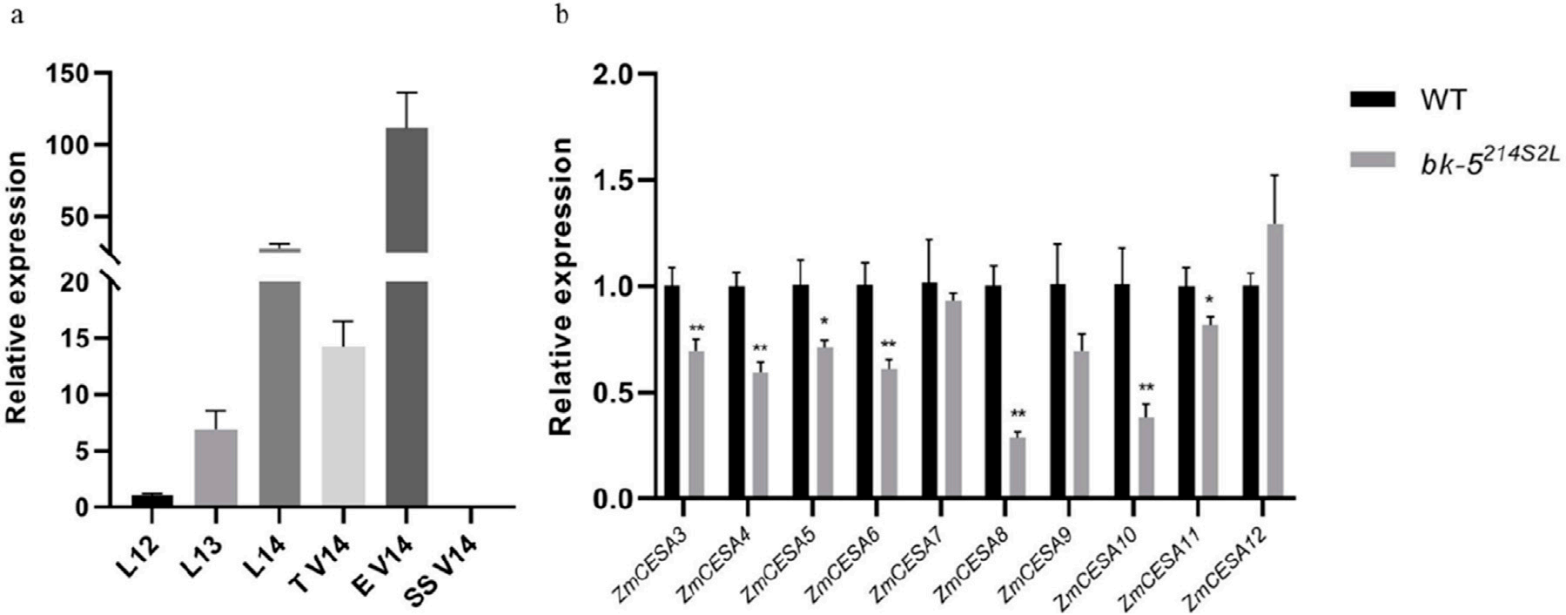
Quantitative real-time PCR analysis results. **(A)** Expression profiling of *bk-5* ^*214S2L*^ in various tissues at the nodulation stage. L, leaf; T, tassel; E, ear; SS, ear stalk. V14 referred to the growth stage. **(B)** Analysis of differential expression of *CesA* genes associated with maize cell wall biosynthesis. The error bars represent SD values, and each sample had three biological replicates.Values are presented as means ± SD, * means *P* < 0.05, ** means *P* < 0.01.

Because mutations in the *bk-5* ^*214S2L*^ gene affected plant brittleness, we investigated its expression in different tissue types of plants at the jointing stage. qRT-PCR results showed that *bk-5* ^*214S2L*^ was most highly expressed in male spikes at the V14 stage (Figure 5A). To understand the expression of *CesA* related to cell wall formation, we performed differential expression analysis of *CesA* involved in the cell wall biosynthesis pathway using male spikes at stage V14 with the highest expression of *bk-5* ^*214S2L*^ as samples (Supplementary Table 3). The expression of all *CesAs* involved in maize cell wall biosynthesis was significantly downregulated (Figure 5B). This indicates that *bk-5* ^*214S2L*^ is essential to primary cell wall and secondary wall biosynthesis in maize.

### 3.6 Evaluation of mutants *bk-5* ^*214S2L*^ and *bk-5* for silage application

**FIGURE 6:**
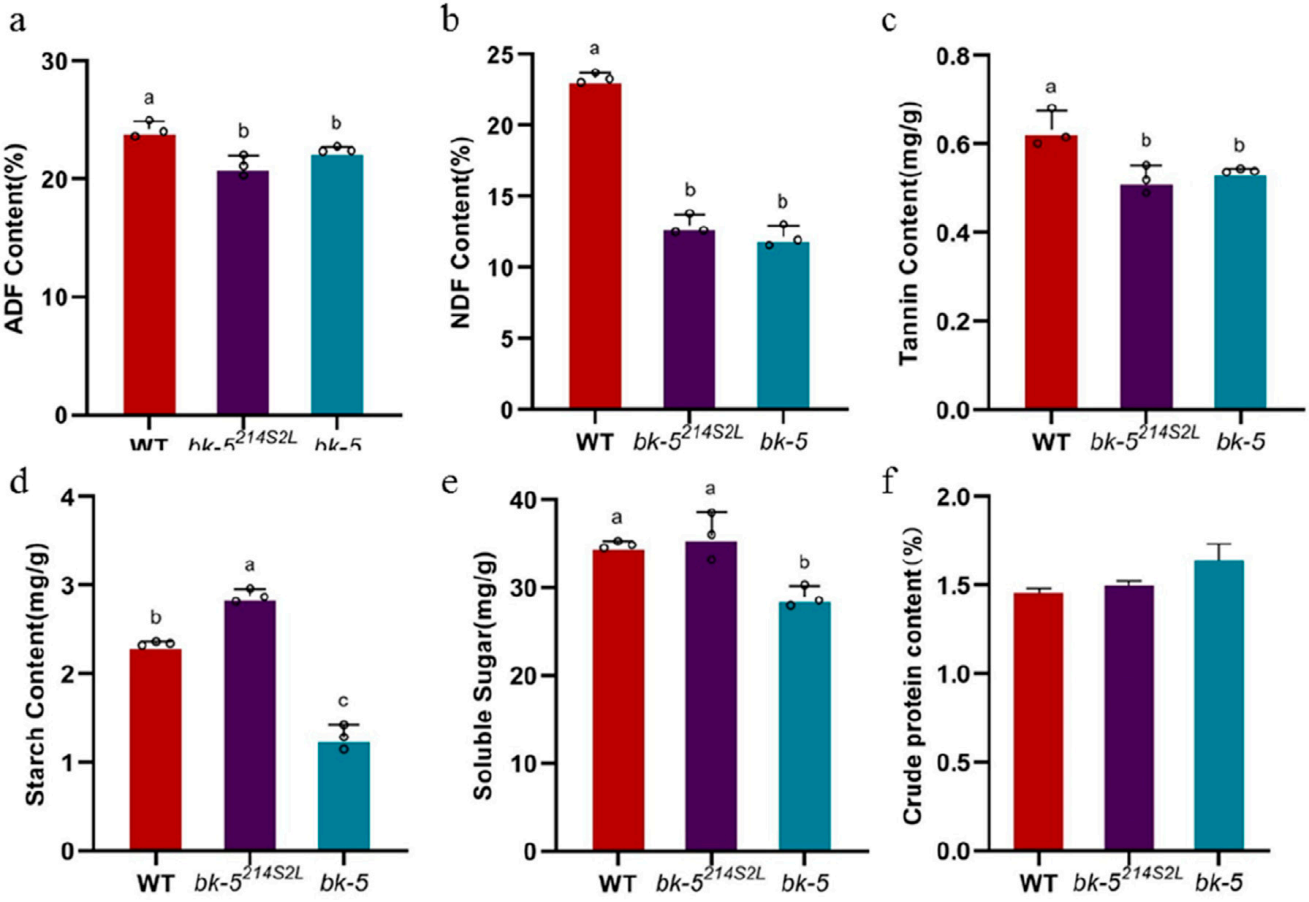
Determination of indicators related to maize silage in stems. **(A)** Acid detergent fiber (ADF) content. **(B)** Neutral detergent fiber (NDF) content. **(C)** Tannin content. **(D)** Starch content. **(E)** Soluble sugar content. **(F)** Crude protein content. Values are presented as means ± SD (Student’s t-test). Different letters **(A–C)** indicate a significant difference between combinations based on two-sided tests (α = 0.05, Kruskal–Wallis test, followed by a Dunn’s *post hoc* test). Three biological replicates were prepared for each group.

The phenotypic properties of mutant fibers provide theoretical guidance for improving the palatability of maize silage; therefore, we determined the relevant indices for RP125, *bk-5* ^*214S2L*^, and *bk-5* silage evaluation, including ADF, NDF, tannin, starch, soluble sugar, and crude protein contents. The ADF percentages in the stems of RP125, *bk-5* ^*214S2L*^, and *bk-5* were 24.23%, 21.15%, and 22.51%, respectively, and the ADF content of *bk-5* ^*214S2L*^ and *bk-5* stems was significantly lower than that of RP125 stems, and the ADF content of *bk-5* ^*214S2L*^ stems was 12.71% lower than that of RP125 stems (Figure 6A). However, the ADF content of *bk-5* leaves was 12.71% lower than that of RP125 leaves. *bk-5* leaves had significantly lower ADF content than RP125 leaves, and ADF content of *bk-5* ^*214S2L*^ leaves was not significantly different from RP125 leaves (Supplementary Figure 6A). The NDF percentage of *bk-5* ^*214S2L*^ stems was 12.98%, which was significantly lower than that of RP125 stems by 44.43%, and the NDF percentage of *bk-5* stems was 12.18%, which was significantly lower than that of RP125 stems by 47.86% (Figure 6B). NDF content was similarly significantly reduced in the leaves of *bk-5* ^*214S2L*^ and *bk-5* compared with RP125 (Supplementary Figure 6B). The detergent fiber contents of both *bk-5* ^*214S2L*^ and *bk-5* complied with the regulations for primary silage maize. The tannin content of *bk-5* ^*214S2L*^ stems was significantly reduced by 17.46% and that of *bk-5* stems was significantly reduced by 14.29% compared with RP125 (Figure 6C). In leaves, no significant difference was found between the tannin contents of *bk-5* ^*214S2L*^, *bk-5*, and RP125 (Supplementary Figure 6C). The starch content of *bk-5* ^*214S2L*^ stems was significantly higher than that of RP125 stems by 22.6%, and the starch content of *bk-5* stems was significantly lower than that of RP125 stems by 45.1% (Figure 6D). The starch content of *bk-5* ^*214S2L*^ leaves was not significantly different from that of RP125 leaves, and the starch content of *bk-5* leaves was significantly reduced by 14.7% compared with RP125 and by 10.2% compared with *bk-5* ^*214S2L*^ (Supplementary Figure 6D). For soluble sugars, the soluble sugar content was not significantly different between RP125 and *bk-5* ^*214S2L*^ stems, whereas the soluble sugar content of *bk-5* stems was significantly lower than that of both RP125 and *bk-5* ^*214S2L*^ stems (Figure 6E); the soluble sugar content of both *bk-5* ^*214S2L*^ and *bk-5* leaves was significantly higher than that of RP125 leaves (Supplementary Figure 6E). The crude protein content of both *bk-5* ^*214S2L*^ and *bk-5* stems and leaves was slightly elevated compared with RP125 (Figure 6F; Supplementary Figure 6F). Thus, based on phenotypic properties and silage-related indices, the weak allelic variant of *bk-5*, *bk-5* ^*214S2L*^, can be used as a high-quality roughage resource to provide sufficient effective fibers for livestock to reduce the risk of rumen acidosis and ensure rumen health of livestock. It can also provide easily digestible NDF and ADF, increase roughage energy, reduce concentrate feed usage, and lower feed cost.

## 4 Discussion

Plant stem development is closely related to many biological processes, including cell wall synthesis, cell wall modification, and vascular bundle formation. These factors directly influence the mechanical strength of straw. The brittle stalk phenotype is associated with several genes (Li et al., 2003; Roudier et al., 2002), which have been identified in many plants and have been cloned in maize. Among these, the typical maize mutants are *bk-2* and *bk-4* (Sindhu et al., 2007; Jiao et al., 2019).

All aboveground plant parts of the maize *bk-2* mutant exhibit high brittleness, and these parts are highly susceptible to breakage upon bending, a phenomenon that occurs at the five-leaf stage (Jiao et al., 2019). Similarly, the *bk-4* mutant is a dwarf plant with weak stems, and all parts of the plant, such as stems, midribs, stilt roots, and male spikes are brittle and break easily in windy conditions. Plants also fall because of stem brittleness and low strength. Hence, both mutant plants require staking throughout their lives for field propagation. These drawbacks create obstacles when studying the growth and development of mutants as well as their applications. Therefore, finding a plant that not only has a brittle stalk but also grows and does not fall as well as identifying brittle stalk genes are important to understand the molecular mechanism of plant brittleness for innovation in the forage industry. The *bk-5* ^*214S2L*^ mutant avoids the shortcomings of *bk-2* and *bk-4*, and is truly brittle. Its brittleness and resistance to fall make it ideal for forage production.

The reduction in cellulose and lignin contents can increase the digestibility of stover, thus increasing the feeding value of the crop, particularly silage maize (Wang et al., 2020; Vinayan et al., 2021). China has a large area under maize cultivation, and if maize stover can be fully used as feed, it can reduce feed production costs and resolve any feed resource shortage. The country also generates 200–300 million tons of maize straw each year. Because of the poor quality and low nutrient content of the straw of maize varieties, the operation cost is high and the nutritional value deteriorates further if it is processed in the pre-industrial stage. Thus, much straw is burnt or left to rot, which is a wastage of resources and pollutes the environment. From this perspective, the brittle stalk and friable straw characteristics of crops are favorable to the feed industry. For breeding applications targeting unfavorable or favorable traits, molecular markers based on brittle stalk genes can be developed for breeding maize such as the dual-purpose maize *bk-5* ^*214S2L*^. Although *bk-5* ^*214S2L*^ has led to improvements in the mechanical strength of the stem, further research is needed before applying this gene in production. We also plan to conduct a Genome-Wide Association Study (GWAS) in combination with other previously reported brittle-stalk-related genes such as *bk2* and *bk4* to screen for excellent haplotypes. Additionally, we will design molecular markers for the genetic improvement of forage maize (Sindhu et al., 2007; Shuping et al., 2019).

## Data Availability

The original contributions presented in the study are included in the article/Supplementary Material, further inquiries can be directed to the corresponding author.
