# Supplementary material for: bk-5 214S2L , an allelic variant of bk-5, as high-quality silage maize genetic resource
Source: Front Genet. 2025 Feb 28;16:1483839. doi: 10.3389/fgene.2025.1483839 (PMC11906420; doi:10.3389/fgene.2025.1483839)
Supplement: Supplementary file 1 [file Table1.docx]

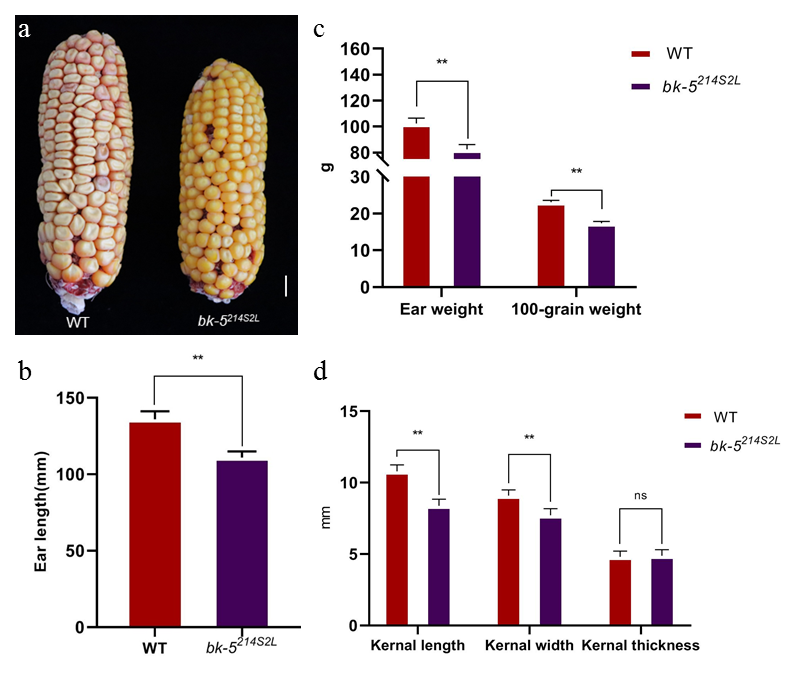


**Supplemental figure 1 | Ear phenotype and agronomic traits wild-type RP125 and mutant *bk-5^214S2L^*.** (a) Phenotypic comparison of *bk-5^214S2L^* and wild-type ears. (b) the agronomic traits analysis of ear length of wild-type and *bk-5^214S2L^*. (c) the agronomic traits analysis of ear weight and 100-grain weight of wild-type and *bk-5^214S2L^*. (d) the agronomic traits of kernel length, kernel width, and kernel thickness of wild-type and *bk-5^214S2L^*. The data was presented as values are given as means ± SD and statistically calculated by Student’s t test. ns, no significant change, and **(P < 0.01) indicate significant differences between wild-type and *bk-5^214S2L^*. The means of ear length, thickness and weight were calculated based on three ears. The means of kernel length, width and thickness were calculated based on ten kernels. 100-grain weight was calculated based on 100 kernels weight with three biological repetitions.


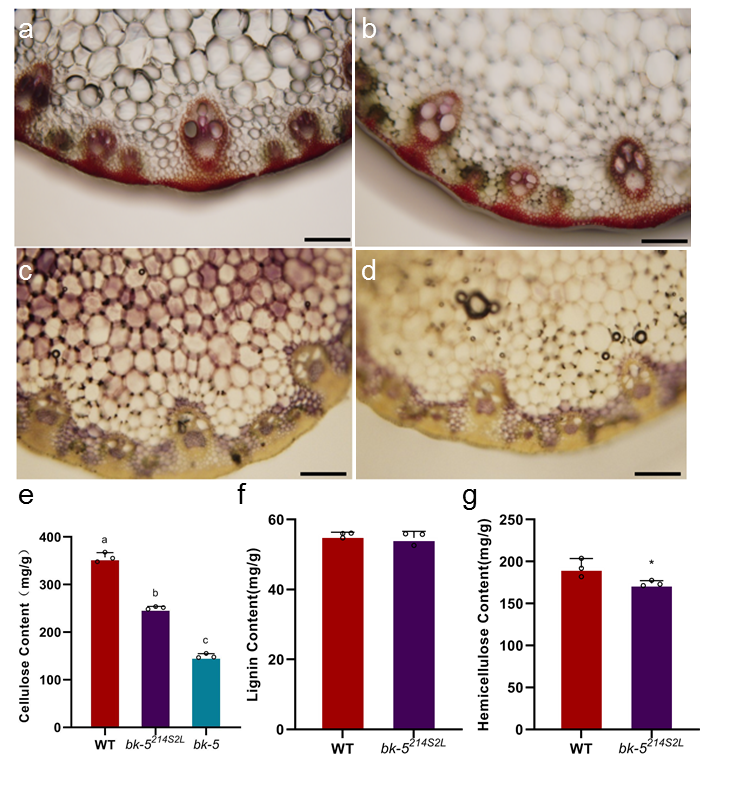


**Supplemental figure 2 | Histochemical staining and cell wall contents of leaf veins.** (a, b) Staining of cellulose and hemicellulose of wild-type (a) vein and *bk-5^214S2L^* (b) vein by zinc chloride iodide solution. The darker the color is, the higher the content of cellulose and hemicellulose is. (c, d) Staining of lignin of wild-type (c) vein and *bk-5^214S2L^* (d) vein by phloroglucinol solution. Pink represents the staining of lignin, and the darker pink the color is, the higher the content is. (e) The content of cell wall cellulose of *bk-5^214S2L^* and wild-type vein. (f) The content of cell wall lignin of *bk-5^214S2L^* and wild-type vein. (g) The content of cell wall hemicellulose of *bk-5^214S2L^* and wild-type vein. All the samples were from the plants at jointing stage. Sections were taken to observe the leaf veins in the spikelet leaves. The data was presented as values are given as means ± SD and statistically calculated by Student’s t test. *(p < 0.05) indicate significant differences between wild-type RP125 and *bk-5^214S2L^*. Three biological repetitions each group were detected.


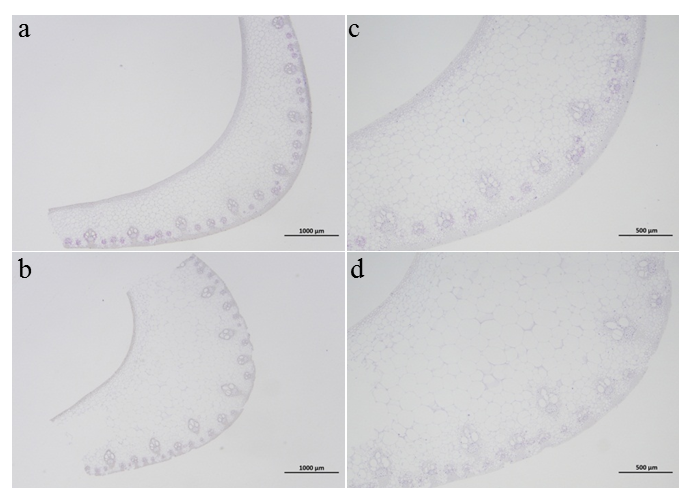
**Supplemental figure 3 | Paraffin sections of** **leaf veins.** (a) 20 times lower wild-type leaf vein; (b) 20 times lower *bk-5^214S2L^* *5* leaf vein; (c) 40 times lower wild-type leaf vein; (d) 40 times lower *bk-5^214S2L^* leaf vein. (a, b) Scale bar: 1000 μm; (c, d) Scale bar: 500 μm.


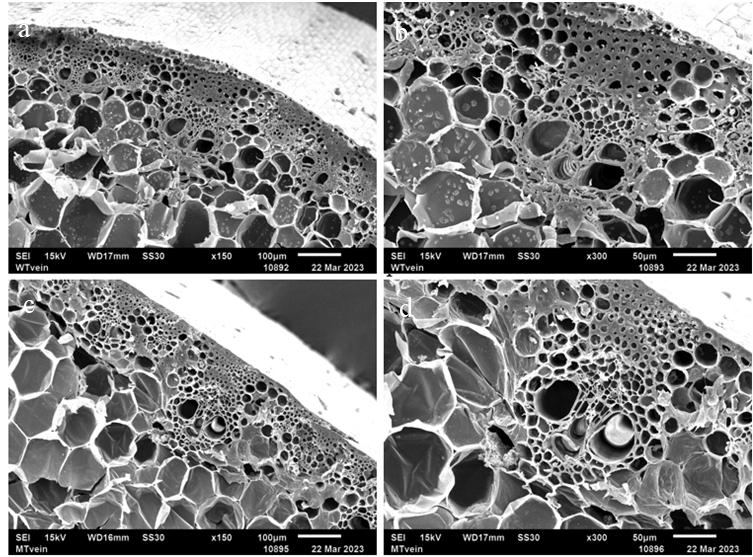


**Supplemental figure 4 | Cross-section under scanning electron microscopy of leaf veins.** (a, b) Cross sections of wild-type leaf veins. (c, d) Cross sections of *bk-5^214S2L^* leaf veins. Different magnifications (150×, 300×) are shown.


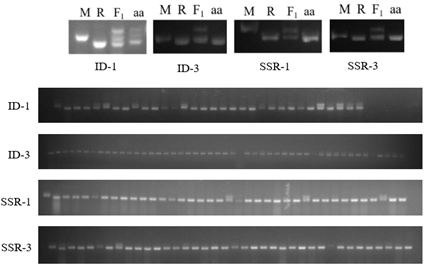


**Supplemental figure 5 | Preliminary screening of linkage region using F2 segregated population mixed by BSA analysis.** Below are pictures of electrophoresis gels of the four selected SSR markers used for BSA analysis. The wild-type and mutant pools were used as DNA templates in lanes 3 and 4 of each gel, respectively; Mo17 and RP125 were used as controls in lanes 1 and 2, respectively, to determine the polymorphism of the molecular markers. M, Mo17; R, RP125; F1, dominant mixed pool; aa, recessive mixed pool.


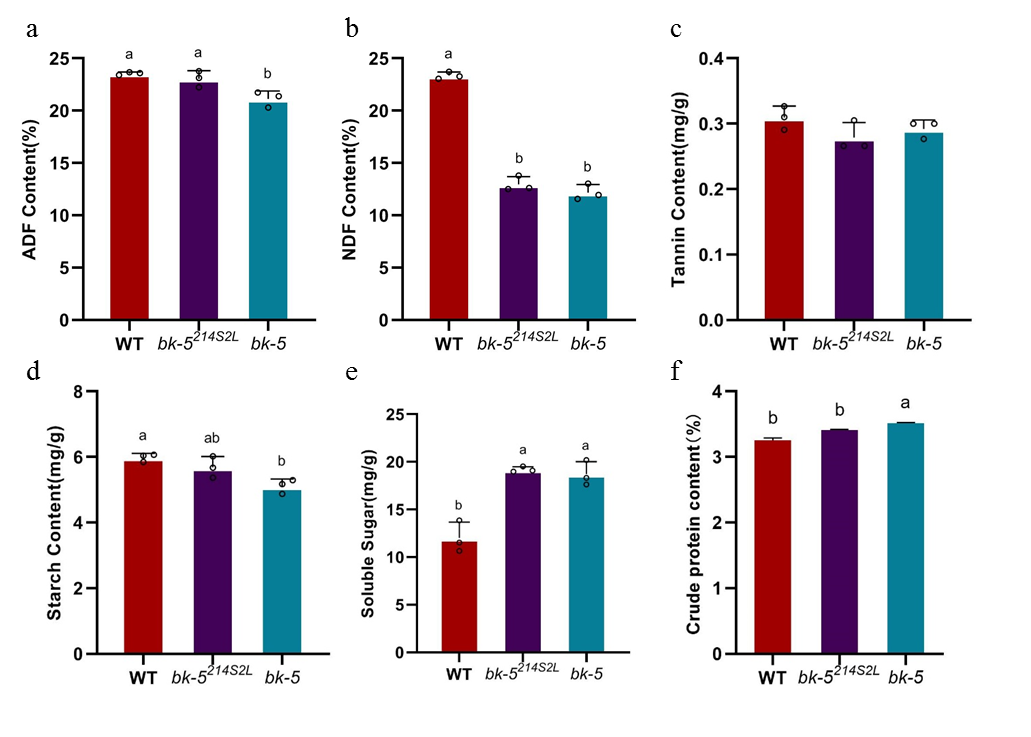


**Supplemental figure 6 | Determination of indicators related to maize silage in leaf.** (a) Analytical results of acid detergent fiber content determination of wild-type, mutant *bk-5^214S2L^* and *bk-5*. (b) Analytical results of neutral detergent fiber content determination of wild-type, mutant *bk-5^214S2L^* and *bk-5*. (c) Tannin content determination analysis results of wild-type, mutant *bk-5^214S2L^* and *bk-5*. (d) Analytical results of starch content determination of wild-type, mutant *bk-5^214S2L^* and *bk-5*. (e) Analytical results of soluble sugar content determination of wild-type, mutant *bk-5^214S2L^* and *bk-5*. (f) Analytical results of crude protein content determination of wild-type, mutant *bk-5^214S2L^* and *bk-5*. The data was presented as values are given as means ± SD and statistically calculated by Student’s t test. Different letters between boxes (b–c) indicate significant differences between combinations based on two-sided tests (α = 0.05, Kruskal–Wallis test, followed by a Dunn’s post hoc test). Three biological repetitions each group were detected.

**Supplemental table 1 | Population segregation of mutan*t bk-5^214S2L^.***

| group | Normal strain | Mutants | Theoretical proportion | χ^2^ value (χ^2^_(0.05,1)_ =3.84) |
| --- | --- | --- | --- | --- |
| Mo17×*bk-5^214S2L^* | 1443 | 441 | 3:1 | 2.464 |

**Supplemental table 2 | Partial molecular markers related to gene location**

| Molecular  Markers | Forward primer | Reverse primer | Position (V4) |
| --- | --- | --- | --- |
| ID-1 | CTATTTCTTCCAGCCAGCCAC | GCAGGTGAGCCACTTAAACA | 3: 199,910,233 |
| ID-3 | GCTCCGTTTTGCCCCTCTAT | AAGCTCTGCGTCTGTCTGTC | 3: 200,440,647 |
| SSR-1 | TTTACCACACGGATGCAATGG | CGCATGCCAGTTACCAGGTG | 3: 201,379,303 |
| SSR-2 | TGCCTGAAAGGTCTCAATCTCAT | TCCAGAGACCAAAGGCACTG | 3: 201,450,434 |

**Supplemental table 3 | Primer list of *CESA* gene RT-qPCR analysis.**

| **Name** | **Sequence** |
| --- | --- |
| ZmCESA3-F | ATGGGGTCCACTATTCGGGA |
| ZmCESA3-R | GAAGCAAGAAGGACGGACCA |
| ZmCESA4-F | GCCCCCATCAATCTTTCGGA |
| ZmCESA4-R | CTCTCCAGGAACTTGAGCCG |
| ZmCESA5-F | CCTCTAAGGCCACTGACGAA |
| ZmCESA5-R | ACCGCTATTGATTGCGTAGGA |
| ZmCESA6-F | GACAACCCTGCTCCTACTGAA |
| ZmCESA6-R | GACCCTTGAGGAACGGGTAAA |
| ZmCESA7-F | CCGTGACATCAAAGGGTGGA |
| ZmCESA7-R | ATCGCATTTGAAACGCCAGC |
| ZmCESA8-F | CGCTCCTCCTGCTGAACTT |
| ZmCESA8-R | CCACCAGACCCTTGAGGAAC |
| ZmCESA9-F | GGACGACACTTCTGATCCCG |
| ZmCESA9-R | TGAACGATCACCCAGAAGGC |
| ZmCESA10-F | CGGGGACCTCTACCTCTTCA |
| ZmCESA10-R | ACGATGCCCACCATGTTGAT |
| ZmCESA11-F | GCCTGCGTGAGAGGGTCAC |
| ZmCESA11-R | TACCAATTCCCGACAAGCTGC |
| ZmCESA12-F | GCTCTACGCCTTCAAGTGGA |
| ZmCESA12-R | GAAGAGCTTGCCGAAGAGGG |
